# Electronic health records for biological sample collection: feasibility study of statin-induced myopathy using the Clinical Practice Research Datalink

**DOI:** 10.1111/bcp.12269

**Published:** 2014-04-22

**Authors:** Helen O'Meara, Daniel F Carr, Jane Evely, Mark Hobbs, Gerard McCann, Tjeerd van Staa, Munir Pirmohamed

**Affiliations:** 1 Department of Molecular and Clinical Pharmacology, Wolfson Centre for Personalised MedicineLiverpool, UK; 2 Clinical Practice Research Datalink, Medicines and Healthcare products Regulatory AgencyLondon, UK; 3 Utrecht Institute for Pharmaceutical Sciences, Utrecht UniversityUtrecht, The Netherlands; 4 London School of Hygiene & Tropical MedicineLondon, UK

**Keywords:** electronic health records, myopathy, research governance, statin

## Abstract

**AIMS:**

Electronic healthcare records (EHRs) are increasingly used to store clinical information. A secondary benefit of EHRs is their use, in an anonymized form, for observational research. The Clinical Practice Research Datalink (CPRD) contains EHRs from primary care in the UK and, despite 1083 peer-reviewed research publications, has never been used to obtain pharmacogenetic samples. Using a statin-induced myopathy paradigm, we evaluated using the CPRD to obtain patient samples for a pharmacogenetic study targeting 250 cases and 500 controls from UK general practitioner (GP) practices.

**METHODS:**

The CPRD identified potential patients fitting specific case-definition criteria (active rhabdomyolysis or creatine phosphokinase > four times the upper limit of normal), and corresponding GP practices were asked to invite patient participation. Consenting patients were requested to provide either saliva or blood samples and to complete an ethnicity questionnaire. Control subjects were recruited from the same GP practice (saliva) or a small number of practices (blood). Samples were forwarded for DNA extraction.

**RESULTS:**

Thirty-six months of recruitment yielded DNA samples from 149 statin-induced myopathy cases and 587 tolerant controls. Data show that contacting patients through their GP is a reliable method for obtaining samples without compromising anonymity. Saliva collection directly from patients was considerably less effective than blood sampling. After 10 months of recruitment, saliva sampling was suspended in favour of blood sampling.

**CONCLUSIONS:**

We demonstrate the potential of EHRs for identifying accurately phenotyped cases and controls for pharmacogenetic studies. Recruitment was successful only because of the willingness of GP practices to participate and the existence of strong doctor–patient relationships. The present study provides a model that can be implemented in future genetic analyses using EHRs.

**WHAT IS ALREADY KNOWN ABOUT THIS SUBJECT**

- Electronic health records, such as the Clinical Practice Research Datalink (CPRD), are a potentially a valuable resource for identifying rare events, such as rare adverse drug reactions.
- To date, no study has used the CPRD to identify and recruit patients to obtain biological samples for the purpose of genetic studies.
- Current UK research governance procedures require research and development approval from each participating subject even in studies that are largely non-interventional.

**WHAT THIS STUDY ADDS**

- This study demonstrates the utility of electronic medical records in identifying well-phenotyped patients with a rare adverse drug reaction (statin-induced myopathy).
- A novel methodology is presented for the recruitment of patients identified in the CPRD to a case–control study.
- The present study highlights the need for streamlining UK research governance procedures in order to facilitate and expedite national studies using patients recruited from multiple sites.

## Introduction

Healthcare systems are increasingly using computers to record and store clinical information. Electronic health records are also valuable for undertaking epidemiological research using anonymized individual patient data. A typical example is the General Practice Research Database, recently renamed as the Clinical Practice Research Datalink (CPRD). The CPRD has evolved over time, with increasing population coverage and, through linkages with secondary care data and laboratory data, provides access to more complete data sets. This makes it a highly valuable resource that is widely used by academic, regulatory and industry sectors. Although the CPRD has been widely used for observational research, the data have not been linked to biological samples, such as DNA. The UK biobank, in contrast, currently has access to biological samples and will eventually have access to linked health records. The CPRD is much larger than the UK biobank and would be particularly valuable to improve our understanding of the genetic basis of rarer phenotypes, if these could be linked to biological samples. One area of medical research that would greatly benefit from this is the pharmacogenetics of adverse drug reactions.

In pharmacogenetic studies, genetic markers (from either candidate genes or the whole genome) from patients who have experienced an adverse drug reaction (cases) can be compared with those from patients who have had no reaction (controls). In order to test the utility of the CPRD for the recruitment of biological samples from patients with adverse drug reactions, we have used statin-induced myopathy as a paradigm. Although highly successful in the reduction of low-density lipoprotein cholesterol and the prevention of coronary heart disease [1–9], statins are also associated with muscle toxicity. Manifestations including pain, raised creatine phosphokinase (CPK) and, rarely, rhabdomyolysis [5,10] are observed with all types of statins and are more prevalent with higher doses. Although the overall frequency of myopathy was found to be low in clinical trials (<5%) [9], the incidence in clinical practice is thought to be much higher (10–15%) [11,12], particularly the incidence of muscle pain without any rise in CPK. A genome-wide association study revealed an association with an *SLCO1B1* variant [13,14]. Such pioneering use of the CPRD would provide a golden opportunity to identify large numbers of affected patients with other adverse drug reactions in the future in order to elucidate the genetic predisposing factors.

## Methods

### Ethical approval and research and development approval

Ethical approval was obtained from the National Research Ethics Committee North West 2 – Liverpool Central, and approval to use the CPRD data was obtained from the Independent Scientific Advisory Committee at the Medicines and Healthcare products Regulatory Agency. Research and development approval was obtained from the host National Health Service (NHS) organization (Royal Liverpool and Broadgreen University Hospitals Trust, Liverpool, UK). In addition, because recruitment was to involve general practitioner (GP) practices from across the UK, it was necessary to obtain local research and development approval from the primary care trusts (PCTs) of practices containing potentially eligible patients. For simplicity, the principal investigator (PI) was also nominated as the local collaborator (LC) for each site. The GP practices from within a PCT were not contacted until site approval was obtained from the medical board. Finally, all participants provided informed consent in accordance with the Declaration of Helsinki. A recruitment target of 250 cases and 500 controls was set.

### Identification of patients through the Clinical Practice Research Datalink

Cases and controls were considered only if they were at least 18 years old and had been prescribed a statin, with the first prescription at least 1 year after the start of CPRD data collection. The first laboratory record of CPK measurement or the first medical record in the electronic health records of rhabdomyolysis in the 3 months following a statin prescription and after 1 January 2000 was identified. As such, all cases were considered to be current users of statins at the date of the CPK measurement or rhabdomyolysis. The index date was the date of the CPK measurement or rhabdomyolysis record. The CPK values were classified according to the upper limit of normal (ULN; defined as 195 units l^−1^ for men and 170 units l^−1^ for women). Cases with myocardial infarction, trauma or falls recorded in the period from 1 month before to 2 weeks after the CPK measurement or rhabdomyolysis were excluded.

### Phenotype definitions

#### Cases: phase I (months 1–10)

Patients with a history of rhabdomyolysis (high CPK levels associated with symptoms and evidence of myoglobin in the urine and often accompanied by renal failure) or a rise in CPK of >10 times.

#### Cases: phase II (months 11–36)

The criteria was relaxed to include patients with a CPK > four times the ULN. Saliva sample collection was halted.

#### Controls

The selection criteria for controls were current statin users (at least 90 days from the start of recruitment) without a history of above-normal CPK values and no history of recorded symptoms of myopathy. For saliva, controls were also recruited from the same practice as their cases during phase I.

The following characteristics were measured at the date of CPK measurement or rhabdomyolysis: age, gender, body mass index, smoking status, number of prescriptions issued in the 3 months before, prescribing in the 6 months before of antihypertensives, drugs interacting with statins through cytochrome P450 3A4 (CYP3A4) (amiodarone, fibrates, cyclosporine, azole antifungals, macrolide antibiotics, protease inhibitors and calcium channel blockers), drugs interacting through mechanisms other than a CYP3A4 interacation (such as digoxin, warfarin, fenofibrate, gemfibrozil and nicotinic acid), oral corticosteroids, medical history of diabetes mellitus, hypothyroidism, hyperthyroidism, chronic obstructive pulmonary disease and records in the 1 month before of myalgia.

### Practice recruitment

The CPRD contacted GP practices by letter inviting them to participate in the study. If willing, they were then asked to review a list of eligible registered patients and remove any they considered unsuitable for the study (due to, for example, non-Caucasian descent). During phase I, the participating practices were randomized to collection of either blood or saliva case samples. During phase II, solely blood samples were requested.

### Patient recruitment

For recruitment of cases, GP practices were contacted by letter and informed of potential cases for the study identified through the CPRD database. All samples were anonymized by the GP using a unique CPRD identifier code such that no patient identifiable information was included with the sample or questionnaire. Each patient letter contained an invitation letter signed by the GP, a patient information leaflet with contact details of the research team and a consent form, as well as a personalized letterhead from the GP practice. Patients wishing to participate were asked to sign the consent form and return it to the practice, who forwarded it back to the CPRD. Those practices asked to collect blood samples made arrangements for patients to attend the practice, whilst practices asked to collect saliva samples sent a supplied saliva collection kit (Oragene DNA collection kit; DNA Genotek, Ontario, Canada) to the patient's home address. All patients were also asked to complete an ethnicity questionnaire, which was sent along with their sample by mail in a pre-addressed envelope to The University of Liverpool.

For the collection of control blood samples, control subjects were selected from a small number of participating practices, whereas for the collection of saliva, control subjects were selected from the same practice as their matched cases.

### Extraction of DNA

Genomic DNA was extracted from both whole blood and saliva using standard automated procedures (Perkin Elmer Chemagen, Baesweiler, Germany) and from saliva using the standard protocol supplied by Genotek. The DNA concentration was measured using the Nanodrop ND-1000 Spectrophotometer (NanoDrop Technologies, Wilmington, DE, USA). For samples obtained through the CPRD, a record of the DNA extraction date was logged onto the Citrix server so that the GPs could be reimbursed for their services.

## Results

### Research approvals

**Figure 1:**
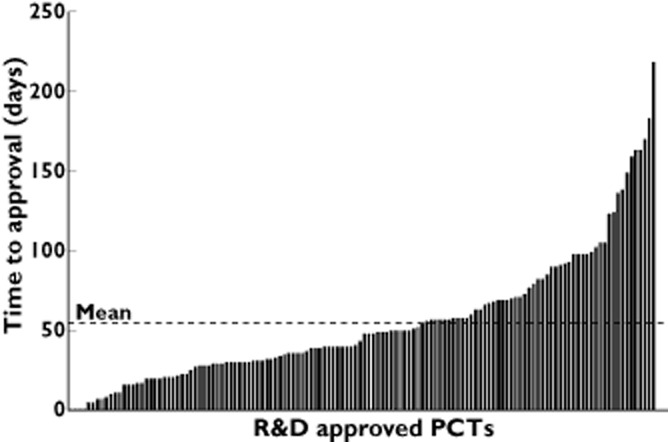
Time taken to obtain local approval in England, Scotland and Wales beginning with submission to National Institute for Health Research (NIHR) Coordinated System for gaining NHS Permission (CSP) through the integrated research application system (IRAS) to receipt of final site approval. Each bar represents time to approval for an individual primary care trust, with the mean time to approval indicated by the dashed line

Individual applications for site approval were sent to 132 PCTs across England, Wales and Scotland. Although ethical and Independent Scientific Advisory Committee approvals were obtained within the required time frames, obtaining local approval was a major challenge from 132 separate sites (Figure 1) and took substantially longer, with the final approval being granted more than 8 months after the process began. The mean ± SD length of approval time was 54.7 ± 40.6 days. Many PCTs raised queries, which included questions about study design (despite the fact that this had received peer-reviewed funding), the content of the patient information leaflets (despite approval from ethics committees), the need for approval from every GP practice instead of the governing PCT (which would have massively increased the bureaucratic burden) and the need to have a local collaborator at every site (despite the minimal nature of the intervention, i.e. a simple blood or saliva collection with anonymization maintained).

### Practice recruitment

During phase I, the CPRD received a positive response from 51% of practices that were contacted to recruit blood case samples and 57% for saliva samples (Table 1). There were a number of reasons for practice non-involvement in the study, including the following: nonresponse; simply declining to participate; willing participation but no suitable patients; and refusal of permission to participate from the primary care trust. From the list of potential myopathy patients provided to practices by the CPRD, 43% were deemed suitable by their GP in phase I (blood and saliva) and 35% in phase II (Table 1). Ultimately, patient samples were received from only 62 of 132 (47%) of the PCTs for which research approval was obtained.

**Table 1:** Recruitment data for practices and patients recruited by the Clinical Practice Research Datalink using both blood and saliva sampling methods

	Phase I‡			Phase II	
	Cases, blood	Cases, saliva	Controls, saliva	Cases, blood	Controls, blood
**Practices**					
** Invited to participate**	109	101	13*	336	54
** Declined to participate**	15	14	0	19	1
** Did not respond**	23	17	1	142	37
** Refused permission to participate**	1	0	0	21	0
** Current patients unsuitable**	16	21	1	27	0
** Recruited (%)**	55 (51)	58 (57)	11 (85)	127 (38)	16 (30)
**Patients**					
** Potential**	241§	241§	101†	953§	3764
** Suitable**	102	121	77	505	1389
** Declined (%)**	14 (14)	46 (38)	0 (0)	123 (24)	1 (<1)
** Awaiting patient response or inadequate sample (%)**	44 (43)	53 (38)	39 (57)	298 (59)	839 (60)
** Adequate sample received (%)**	43 (42)	22 (24)	38 (43)	84 (17)	549 (30)

* Practices had previously obtained case saliva samples.

† Patients were age and gender matched to saliva cases.

‡ Phase I data include all saliva samples regardless of date of receipt and case blood samples received up to 31 March 2011.

§ Total includes all patients on the Clinical Practice Research Datalink fulfilling myopathy criteria (creatine phosphokinase > 10 times the upper limit of normal for phase I and > four times the upper limit of normal for phase II) from recruited and nonrecruited practices.

**Figure 2:**
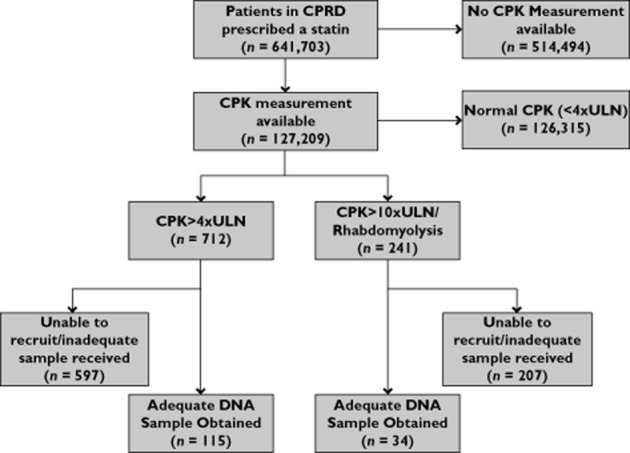
Schematic diagram showing the total number of potential statin-induced myopathy patients within the Clinical Practice Research Datalink (CPRD) and the numbers fulfilling the inclusion criteria who were ultimately recruited into the study

From a total of 953 patients identified within the CPRD who fulfilled the criteria for statin myopathy, we were able to obtain adequate DNA samples from 149 (Figure 2), representing a recruitment rate of 15.6%. Of the 241 cases with severe myopathy (CPK > 10 times the ULN/rhabdomyolysis), we were able to obtain adequate samples from 32 (13.3%).

### Patient recruitment and biological sample receipt

**Figure 3:**
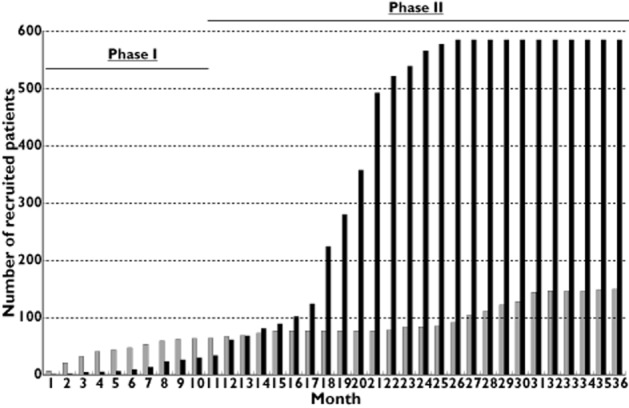
Cumulative recruitment of statin-induced myopathy cases and controls (*n* = 736) over the 36 month period of study recruitment. Data are based on the date on which a valid biological sample was received by the laboratory and include only viable patients. 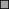, cases; ▪, controls

During the period from June 2010 and April 2013, a total of 754 patients, with accompanying biological sample received, were recruited (Table 1). Of the 754 samples received into the laboratory by post, 18 were excluded for either sample identification reasons (mislabelling of blood tubes; *n* = 10) or sample quality issues [low volume of sample (*n* = 3) or poor DNA yield/quality (*n* = 1)]. A further four case samples were excluded because closer inspection of phenotype identified them as not fulfilling inclusion criteria. One hundred and thirteen cases were excluded due to data extraction quality control issues, which categorized individuals with an empty data field CPK value as cases. These individuals, however, fulfilled the criteria for controls and were subsequently reclassified. Thus, in all, 736 individuals satisfied the sample and phenotype criteria of cases (*n* = 149) or controls (*n* = 587). Recruitment progress over the 36 month period is shown in Figure 3. Comprehensive phenotype data were obtained for all included patients. A summary of clinical and demographic variables of the cohort are listed in Table 2. We have also been able to replicate the association between statin-induced myopathy and the *SLCO1B1* genetic polymorphism [15] previously described by Link *et al*. [13], providing proof of concept for our approach to case identification.

**Table 2:** Summary of clinical and demographic data from cases and controls from whom a viable DNA sample was obtained

Variable	Missing data (*n*)	Controls (*n* = 587)	Cases (*n* = 149)
**Statin at index**			
** Simvastatin**		344 (59%)	98 (66%)
** Atorvastatin**		175 (29%)	34 (23%)
** Rosuvastatin**		29 (5%)	9 (6%)
** Fluvastatin**		11 (2%)	2 (1%)
** Pravastatin**		26 (4%)	6 (4%)
** Ceruvastatin**		2 (<1%)	0 (0%)
** Mean daily dose [mg day^−1^ (SD)]**		30.9 (+15.9)	31.2 (+19.0)
Mean age [years (SD)]*		69.7 (9.3)	66.2 (10.6)
** Gender**		64% male, 36% female	72% male, 28% female
Mean body mass index*	66 (9%)	28.8 (+5.4)	29.5 (+5.0)
**Smoking status**	36 (5%)		
** Nonsmoker**		237 (42%)	61 (43%)
** Ex-smoker**		238 (43%)	64 (45%)
** Smoker**		83 (15%)	17 (12%)
**Comedications in 6 months prior to index**			
** Antihypertensives**		479 (82%)	108 (73%)
CYP3A4 inhibitors*		72 (12%)	20 (13%)
** Known statin interactor (non-CYP3A4 substrate)**†		51 (9%)	11 (7%)
** Oral corticosteroids**		23 (4%)	4 (3%)
**Occurrence in previous 6 months or 2 weeks after index**			
** Cramps**		5 (1%)	3 (2%)
** Myocardial infarction**		5 (1%)	1 (<1%)
** Renal failure**		16 (3%)	5
** Trauma**		1 (<1%)	1 (<1%)
** Falls**		2 (<1%)	0 (0%)
**Previous history (any time prior to index)**			
** Type 2 diabetes**		154 (26%)	45 (30%)
** Alcohol dependence**		26 (4%)	6 (4%)
** Asthma**		75 (13%)	20 (13%)
** Atrial fibrillation**		61 (10%)	13 (9%)
** Chronic obstructive pulmonary disease**		41 (7%)	7 (5%)
** Hypertension**		382 (65%)	73 (49%)
** Hyperthyroidism**		10 (2%)	3 (2%)
** Hypothyroidism**		47 (8%)	14 (9%)

* CYP3A4-interacting comedications were amiodarone, cyclosporine, azole antifungals, macrolide antibiotics, protease inhibitors and calcium channel blockers.

† Non-CYP3A4-interacting comedications recorded were fenofibrate, gemfibrozil, digoxin, warfarin and nicotinic acid.

## Discussion

This study has demonstrated that the CPRD can be used for the identification and recruitment of patients where there is a need to donate a biological sample. However, there is a huge governance burden that represents a major challenge.

A strength of the study was to use a rare adverse drug reaction, statin-induced myopathy, as the paradigm; this enabled the recruitment of 149 cases of statin-induced myopathy and 587 drug-exposed controls. However, the use of CPK elevation as an inclusion criterion led to initial misclassification of 113 individuals (where a blank data field was incorrectly taken to indicate an elevation) for whom biological samples were received between months 5 and 29. A second line of quality control at the data extraction stage would have prevented this, and was implemented for the remaining recruitment period. It was, however, possible to re-assign these patients as controls. We did not achieve our target of 250 cases, probably due to several reasons, including the governance burden, the difficulties in identifying the right patients in the database, the fact that we concentrated only on elevations in CPK levels, and the lack of willingness of all practices (53%) to take part in the research.

A total of only 14 of 754 samples (1.8%) received were excluded for reasons directly related to the general practice physician or patient's handling of the samples (either mislabelled tube (*n* = 10) or poor sample quality (*n* = 4). Handwritten transcribing of complex alphanumeric patient codes by the physician accounted for the majority of issues. This was addressed later in the study by providing printed labels.

Only 1 of 60 (1.6%) saliva samples provided directly by patients without physician contact was omitted due to poor DNA yield, suggesting that patients were capable of providing sufficient sample without clinical supervision. However, the lack of the reassurance of providing a biological sample directly to a clinician is likely to be a key reason for the decline rate for saliva samples (38%) compared with blood samples (14%) in recruitment phase I. Given the higher ‘success rate’ of blood sample recruitment, we concentrated only on recruitment via blood donation.

The lack of a central, nationalized healthcare research and development approval system meant that 132 individual local primary care provider approval applications had to be processed. This caused significant delays to the study recruitment phase, given that the mean time from application to approval was 54.7 days and the longest time for a single approval was 218 days. The governance burden placed on researchers in the UK has been highlighted by other studies [16,17]. This led to the review of research governance by the Academy of Medical Sciences [18], and has led to the setting up of the Health Research Authority in the UK. A single, streamlined approval process would considerably reduce the administrative burden and lead time for performing studies using electronic health records in multiple sites, but whether this can be achieved is unclear.

Using the recruitment methodology outlined, we were able to recruit a significant number of cases of stain-induced myopathy (*n* = 149). However, this figure represents merely 15.6% of the potential cases available. Although many of the remaining cases may have proved to be unsuitable, it is possible that, with streamlined governance procedures, many of these ‘missed opportunities’ could have been taken and the recruitment targets met.

### Conclusions

The importance of electronic health record databases for conducting research has been widely acknowledged; to date, they have mainly been used for epidemiological research. A case in point is the CPRD, which has resulted in 1083 peer-reviewed publications (as of June 2013). However, such population databases may also serve as a time-and cost-efficient method for identifying patients with rare disorders, including those caused by adverse drug reactions. Our study using statin myopathy as a paradigm of a rare adverse event clearly shows that CPRD can also be configured to obtain significant numbers of viable biological samples for the purpose of pharmacogenomics studies. Given that rare adverse drug reactions have a significant genetic predisposition [19], the use of CPRD and other databases has the potential to revolutionize research in this field.

## Competing Interests

All authors have completed the ICMJE uniform disclosure form at http://www.icmje.org/coi_disclosure.pdf and declare: no other support from any organization for the submitted work; no financial relationships with any organizations that might have an interest in the submitted work in the previous 3 years; no other relationships or activities that could appear to have influenced the submitted work.

This study was funded by a grant from the e-Health Initiative funded jointly by the Medical Research Council (reference: MC_qA137929), Wellcome Trust, EPSRC and ESRC. MP is a National Institute for Health Research (NIHR) Senior Investigator. The authors wish to thank the contributors to the CPRD, recruiting general practice physicians and patients who participated in the study.
